# Pathophysiology of Post-Traumatic Trigeminal Neuropathic Pain

**DOI:** 10.3390/biom12121753

**Published:** 2022-11-25

**Authors:** Olga A. Korczeniewska, Divya Kohli, Rafael Benoliel, Sita Mahalakshmi Baddireddy, Eli Eliav

**Affiliations:** 1 Center for Orofacial Pain and Temporomandibular Disorders, Department of Diagnostic Sciences, Rutgers School of Dental Medicine, Rutgers, The State University of New Jersey, Room D-837, 110 Bergen Street, Newark, NJ 07101, USA; 2 Department of Oral and Maxillofacial Surgery, Nova College of Dental Medicine, Nova Southeastern University, Davie, FL 33328, USA; 3 Unit for Oral Medicine, Department of Oral and Maxillofacial Surgery, Division of ENT, Head and Neck and Oral and Maxillofacial Surgery, Sourasky Medical Center, Ichilov, Tel Aviv 64239, Israel; 4 Rutgers School of Dental Medicine, Rutgers, The State University of New Jersey, Newark, NJ 07101, USA; 5 Eastman Institute of Oral Health, University of Rochester Medical Center, Rochester, NY 14620, USA; 6 Qassim Dental Hospital, Unaizah 56461, Saudi Arabia

**Keywords:** neuropathic pain, trigeminal nerve injury, pathophysiology, trauma

## Abstract

Trigeminal nerve injury is one of the causes of chronic orofacial pain. Patients suffering from this condition have a significantly reduced quality of life. The currently available management modalities are associated with limited success. This article reviews some of the common causes and clinical features associated with post-traumatic trigeminal neuropathic pain (PTNP). A cascade of events in the peripheral and central nervous system function is involved in the pathophysiology of pain following nerve injuries. Central and peripheral processes occur in tandem and may often be co-dependent. Due to the complexity of central mechanisms, only peripheral events contributing to the pathophysiology have been reviewed in this article. Future investigations will hopefully help gain insight into trigeminal-specific events in the pathophysiology of the development and maintenance of neuropathic pain secondary to nerve injury and enable the development of new therapeutic modalities.

## 1. Introduction

Peripheral nerve injuries can lead to pain persisting beyond the normal healing period or beyond the resolution of damage. The sensory innervation to the head and neck region is supplied primarily by the trigeminal nerve. Therefore, neuropathic pain in the orofacial territory can occur as a result of injury to this nerve. Neuropathic pain (NP) has been re-defined by the Neuropathic Pain Special Interest Group (NeuPSIG) of the International Association for the Study of Pain (IASP) as “pain arising as a direct consequence of any lesion or disease affecting the somatosensory system” [1]. The ICOP (International Classification of Orofacial Pain), 1^ST^ edition describes post-traumatic trigeminal neuropathic pain (PTNP) as “a unilateral or bilateral facial or oral pain following and caused by trauma to the trigeminal nerve(s), with other symptoms and/or clinical signs of trigeminal nerve dysfunction, and persisting or recurring for more than 3 months” [2]. PTNP may result from a major craniofacial/oral trauma or may be subsequent to relatively minor dental treatments such as iatrogenic injuries [3]. Traumatic injuries to the trigeminal nerve largely result in either no residual deficit or in a non-painful neuropathy, and only a minority of cases develop into a painful neuropathy. Estimates of the prevalence of PTNP are lacking, which is partly due to changing diagnostic terms and criteria. In general, approximately 3% of patients with trigeminal nerve injuries develop PTNP [4]. PTNP has been shown to negatively impact patient’s quality of life and wellbeing as it interferes with a variety of social functions and daily activities [5]. Patients with severe pain demonstrate elevated levels of depression, pain catastrophizing, and substantially reduced quality of life and coping efficacy levels [5]. Therefore, similar to other chronic pain conditions, PTNP has been associated with a substantial psychosocial burden [6]. The aim of this review is to present some of the peripheral pathophysiology of painful trigeminal neuropathies based on findings from animal research. Injury to the nerve can occur at various parts along the course of the nerve. Pain induced by injury to the peripheral nerve is considered a peripheral neuropathy; in the ganglion, ganglionopathy; in dorsal root, radiculopathy; and in the central nervous system, a central neuropathy. It is important to note that injury anywhere along the course of the nerve may affect other parts of the nervous system. The majority of the current pain research focuses on the spinal nerves and not the trigeminal system. Despite some similarities, it is shown that the pathophysiology of trigeminal nerve is not identical to spinal nerves. Future research focused on trigeminal system is essential to understand the complexities in the pathophysiology of this condition, enabling development of newer, more effective management therapies.

Nerve-injury-induced neuropathic pain involves a multidirectional cascade of complex interactions between neuronal and non-neuronal cells in the peripheral and central nervous systems, leading to peripheral and central sensitization. Therefore, it is important to emphasize that the pathophysiology of PTNP involves a complex communication between the peripheral and central nervous systems that occur in tandem and may often be co-dependent. Although central events play a crucial role in the development of PTNP, due to their complexity, this review will focus on the peripheral mechanism underlying the development of PTNT and will briefly discuss some of the clinical features. The central cascade of events contributing to trigeminal neuropathic pain is outside the scope of this review.

## 2. Causes of Post-Traumatic Trigeminal Neuropathic Pain (PTNP)

Post-traumatic trigeminal neuropathic pain (PTNP) can result from injuries to the trigeminal nerve caused by craniofacial trauma or iatrogenic injuries such as implant therapy, endodontic treatment, third molar extractions or local anesthetic injections [7,8,9,10,11,12,13,14,15,16]. The severity of nerve injuries can range from mild to severe. Macro trauma in the oral and craniofacial region is the most common PTNP etiology [17,18]. Unlike the spinal system, which is more prone to developing NP, injury to trigeminal nerve branches such as tooth extraction, root canal treatment and other iatrogenic injuries more rarely result in chronic NP [15,19]. For example, clinically diabetic neuropathy is a common source of NP in the limbs and trunk with classical signs of paraesthesia and allodynia. In the trigeminal system, the effects are not pronounced and are rarely reported as typical NP [20]. Additionally, pain syndromes such as migraine, cluster headache and trigeminal neuralgia exist only in the trigeminal system. Furthermore, sympathetic nerve sprouting has been observed in the DRG but not TG following peripheral nerve damage [21]. Finally, distinct gene expression changes have been reported in the ganglia of rats exposed to corresponding peripheral nerve injuries [22]. These findings strongly indicate that although the spinal and trigeminal systems share some common characteristics, they differ, particularly in the way they respond to injury [23]. However, due to the relatively limited number of foundational studies on trigeminal nerve injury we have to rely at least in part on studies that are focused on the spinal nerve, assuming that in spite of the differences, the basic processes are similar. As mentioned above, most traumatic injuries to the trigeminal nerve do not develop into a painful neuropathy. Approximately 3.3% of patients with facial fractures will develop chronic NP [17]. The incidence of chronic NP following implant therapy is not clear, but suggested to be less than 8%, common/non-surgical endodontic treatment between 3–13 % and surgical endodontic therapy approximately 5% [3,14,15,24,25,26,27,28,29,30,31,32].

## 3. Clinical Considerations in PTNP

PTNP is characterized by wide inter-individual variability in clinical presentation following similar nerve injuries. This can be only partially explained by genetic, environmental and psychosocial factors. Variation in PTNP clinical characteristics may include the location and the pattern of the injury. It can be limited to the area of injury or spread across dermatomes, and it can be localized or diffused. The pain intensity may vary as well, from mild to severe. PTNP is often described by the patients as burning or shooting, and it is usually continuous, lasting most of the day and on most days [6,33,34]. A limited number of patients report paroxysmal pain that can be spontaneous or initiated by function or touch.

The reported PTNP age of onset is at the 5th decade of life, with a female predominance [6,34,35,36]. The exact mechanism leading to sex differences in pain and analgesia is not fully understood. Painful neuropathies usually present with somatosensory signs or symptoms, which may be negative (e.g., hypoesthesia, hypoalgesia, or anesthesia) and/or positive (hyperalgesia, allodynia, or dysesthesia) as well as with spontaneous and/or evoked pain [6,34]. PTNP patients can also report sensory disturbances such as sensitivity to hot or cold, flushing feeling of swelling or presence of a foreign body that cannot be verified by examination or imaging [33,34]. It has been suggested that the variation in PTNP clinical presentations may be also related to altered activity of different nerve fibers, caused by pathophysiological complexities of sensory processing in nerve trunk axonal damage or perineural inflammation [37,38,39] as well as to alteration in the patients’ ability to modulate pain [40].

Additionally, PTNP has been reported to be associated with a substantial psychosocial burden [6], increased level of depression, pain catastrophizing, as well as reduced quality of life (QoL) and coping efficacy levels [5]. Pain intensity level was shown to be a significant predictor of all mentioned above modalities [5]. The prognosis of PTNP is poor, significant improvement is reported only by 10–20% of the patients and in long-term follow up appointments 30% of the patients report some level of improvement while 50% of the patients report worsening or no change in their pain levels [6].

## 4. Peripheral Events in the Pathophysiology of PTNP

A cascade of events in the nervous system function is involved in the pathophysiology of pain following nerve injuries. The current understanding of these events is primarily based on the findings from animal research that focused predominantly on injuries to the spinal nerves with limited number of studies investigating trigeminal nerve injuries induced neuropathies. The events following nerve injury are time-dependent, progressing from the peripheral to the central nervous system and include changes in the functional, biochemical and physical characteristics of neurons and glial cells [39,41,42]. As mentioned earlier, this article will focus mainly on peripheral events contributing to the pathophysiology of PTNP, however, both peripheral and central processes play a role in the neuropathy development and occur in tandem.

### 4.1. The Role of Receptors and Inflammatory Soup

**Figure 1:**
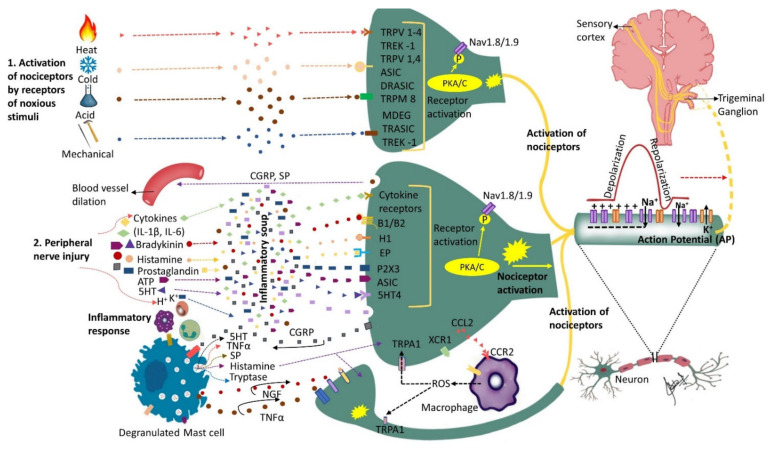
Nociceptors activation and peripheral sensitization: Nociceptors are a specialized class of primary afferents that respond to noxious stimuli. Some nociceptors are activated by noxious stimuli (heat, cold, mechanical or chemical) whereas others respond to substances that are released following tissue injury (cytokines, bradykinin, histamine, endothelin-1, ATP, prostaglandins, etc.). The K+ ions released by neighboring injured cells directly depolarize nociceptor membranes. Trunsduction of noxious stimuli (thermal, mechanical or chemical) into an electric potential or binding of the inflammatory mediators secreted by the injured cells to their corresponding receptors sets off a chain of events leading to nociceptor sensitization and peripheral sensitization. Peripheral sensitization results in lower activation threshold and higher firing rate. Upon activation, nociceptors release substance P and calcitonin-gene-related peptide (CGRP) that initiate inflammatory response including blood vessel dilation and mast cell degranulation (activation). Activated mast cells release histamine, serotonin, nerve growth factor (NGF), substance P (SP) and tumor necrosis factor alpha (TNF-α) that bind to their corresponding receptors on the nociceptor, further sensitizing the nociceptor. Activated nociceptors generate an electric potential. Nerve injury induces further molecular changes, including an increase in sodium (Na+) channels and a decrease in potassium (K+) channels further sensitizing the nociceptor and triggering ectopic activity, as well as alterations in the trigeminal ganglion. Additionally, injured nerves release chemokine CCL2 that attracts macrophages to the site of injury further promoting neuropathic pain development. This figure illustrates only the ascending pathway and not additional pathways that may contribute to the input from the peripheral to the central nervous system.

Nerve injury is accompanied by a release of mediators such as bradykinin, prostaglandin E2, serotonin (5HT), ATP and H^+^ that trigger their corresponding receptors that activate intracellular processes via protein kinase A and C (Figure 1). The activated protein kinases phosphorylate transducer channels, leading to a reduction of thresholds and enhanced excitability of membrane on the peripheral terminals [43,44,45]. The phosphorylation of voltage gated sodium channels (Nav1.8 and Nav1.9) produces enhanced currents and hyperexcitability characterized by reduction of activation threshold and an increased firing rate [46,47,48,49]. Injury to the trigeminal nerve has also been shown to cause a reduction in voltage gated K+ channels (transient and sustained: I_A_ and I_K_) current in the trigeminal ganglion neurons. This causes an increase in the spike frequency and duration and decrease in activation threshold [50,51]. Nerve injury secondary to trauma or disease initiates inflammatory processes, alters electrical activity of neurons, and allows for cross talk between neurons resulting in phenomenon called peripheral sensitization. The substances released during tissue injury initiate a cascade of events that may lead to nociceptors’ activation sometimes even in the absence of a noxious stimulus.

### 4.2. The Role of Chemokines

Chemokine are key players in pain modulation in the peripheral and central nervous systems [52]. Their role in trigeminal pain and their pivotal role in PTNP development has been reviewed recently elsewhere [53]. CCL2 is a proinflammatory chemokine that recruits monocytes, memory T cells and dendritic cells to the site of inflammation [54] that is also known to be a glial cells mediator [55]. It is released from damaged nerves [56] as a direct consequence of the early afferent activity which is critical for the development of neuropathic pain [57]. Released CCL2 promotes increase in Nav1.8 sodium channel activity in primary sensory neurons through a Gβγ-dependent mechanism [58]. Additionally, released CCL2 attracts macrophages to the site of injury [59] by binding to its receptor (CCR2) on macrophages. Macrophages have been shown to promote neuropathic pain development by neuro-immune interactions and their absence was shown to prevent the development of neuropathic pain [60,61]. Other chemokines such as, CXCL13 and its receptor CXCR5 have been implicated in trigeminal neuropathic pain induced by nerve injury [62]. CXCL13 has been shown to contribute to orofacial neuropathic pian via CXCR5/ERK-mediated proinflammatory cytokine production [62] as well as via activation of p38 MAPK in the trigeminal ganglion of mice [63]. Furthermore, the findings from infraorbital nerve injury studies in rats support a direct role for CXCL2 chemokine in trigeminal pain [64]. Inhibition of this cytokine results in the attenuation of mechanical allodynia to levels similar to sham-treated animals [64]. CXCL10 and its receptor CXCR3 is and additional chemokine that has been suggested to have a role in pain. It has been shown that CXCL10 acts on CXCR3 to induce ERK and AKT activation in TG neurons and contributes to the maintenance of trigeminal neuropathic pain in mice [65]. These findings suggest that pronociceptive effects of CXCL10 could act through ERK and AKT signaling.

### 4.3. Peripheral Sensitization

The state of heightened sensitivity in the peripheral tissues is termed as peripheral sensitization and is reflected by a reduction in activation threshold and elevation of nociceptive response [66]. Hyperalgesia (increased pain response to a noxious stimulus) and allodynia (pain response to non-painful stimuli) are among the characteristic features of peripheral sensitization. Some of the common clinical findings in PTNP can be explained by phenomenon of peripheral sensitization. It has been demonstrated that peripheral sensitization is associated with release of inflammatory mediators such as TNF-alpha, IL6, and IL-1 beta, alterations in receptors such as TRPV1, NGF, P2X, P2Y and neurokinin, as well as the release of CGRP and glutamatergic neurochemicals [67] (Figure 1).

### 4.4. Spontaneous Neural Activity

When a portion of the axon or cell body become sufficiently hyperexcitable, it can generate a spontaneous activity (SA) along the nociceptive path at level of axon and/or cell bodies in the sensory ganglia [68,69,70,71,72]. This process is called ectopic activity and is reported to be of two different types, Type 1 SA in the presence of subthreshold membrane potential oscillation and Type 2 in the absence of subthreshold membrane potential oscillation [68]. Within the ganglia, there are two types of conductance: a voltage-dependent, physically activating sodium conductance; and a passive, voltage-independent conductance due to potassium leak. Ectopic firing occurs when the conductance creates membrane potential oscillations and when oscillation sinusoids reach threshold amplitude [73,74,75]. These features of nerve injury may contribute to spontaneous pain seen in patients with PTNP. Inflammation around a nerve with no axonal nerve damage can become a source of pain by elevating spontaneous activity and inducing mechanosensitivity [37,76,77]. If inflammation persists, secondary nerve damage may ensue.

### 4.5. SA in Different Nerve Fibers

Animal as well as clinical electrophysiological studies demonstrate that during SA associated with painful neuropathies, an alteration of firing properties of Aβ, Aδ and C fibers occurs [70,78,79,80,81]. Spontaneous burning or sharp pain may arise from the spontaneous activity in C and Aδ fibers, whereas spontaneous activity in Aβ fibers would drive paresthesia and dysesthesia commonly present in neuropathies [82]. Animal studies show that the bulk of spontaneous activity during onset of pain is due to the spontaneous activity in Aβ fibers, and that there is a direct contribution in spontaneous and evoked pain by spontaneous activity in large diameter myelinated afferents that normally signal touch and vibration [83]. In pathologic conditions, a phenotypic switch (changing electrical characteristics and the expressed neurotransmitters complement) occurs in the Aβ fibers and they become nociceptive, acting as a source of neuropathic pain [83]. Previously injured Aβ fibers, when stimulated, were shown to induce c-fos expression, [84,85,86,87]. These findings suggest that activity in injured Aβ fibers may both drive pain and trigger central sensitization.

### 4.6. Ion Channels Alterations

Nerve injury causes variations in several ion channels in particular their expression, and function. The relative depolarization of resting potential associated with SA may be driven by the enhanced persistent sodium channel currents or reduced potassium leak currents which are active around the resting potential [82]. Hyperpolarizing cationic currents (I_h_) contribute to hyperexcitability of sensitized peripheral fibers. In peripheral nerve injury, the rate of firing and current density of I_h_ current increases in the sensory ganglia and trigeminal ganglion [88,89,90,91,92,93,94]. Mechanical allodynia and ectopic discharge following nerve injury is reportedly reduced by blocking these currents [88,95]. The number of small-diameter sensory neurons positive for T-type Ca^2+^ channel increases due to the inflammatory mediators, in particular bradykinin and adenosine triphosphate (ATP), released during nerve injury and inflammation [96]. These neurons regulate peripheral sensory neuron excitability resulting in an increase in voltage gated Ca^2+^ channel currents in sensory neurons which leads to hypersensitivity [97,98,99,100,101]. In animal models of pain, both neuropathic and inflammatory, a silencing of Cav3.2 demonstrate an anti-nociceptive effects and pharmacological inhibitors of T-type Ca^2+^ channels have been shown to have analgesic effects [100,102,103,104,105,106,107,108].

### 4.7. The Role of Neurovascular Interactions in Orofacial Neuropathic Pain

Peripheral and central changes induced by nerve injury are mediated by the complex interactions between multiple cell types including neurovascular units (neurons, glial cells, endothelial cells and immunocytes) that are responsible, in part, for regulating the homeostasis of the blood–nerve barrier (BNB) [109]. Infraorbital nerve injury may lead to local disruption of the BNB and therefore increased vascular permeability. These changes result in increased infiltration of immunocytes and promote local neuroinflammation as indicated by a significant upregulation of immunocytes (CD3, CD11b) and innate immunity receptors (Toll-Like Receptor 2 (TLR2) and 4 (TLR4)) mRNA within the ION parenchyma [110,111] and neuroimmune interactions [112]. In rats exposed to infraorbital nerve chronic constriction injury, infraorbital nerve BNB disruption was linked to Hedgehog signaling and TLR4-mediated pathways [113]. Released harmful molecules such as IL-1B and chemokine MCP-1 [110] as well as immunocytes contribute to nerve sensitization and neuropathic pain [113]. Hedgehog signaling and its main effector Sonic Hedgehog (SHH) control and regulate a repair program essential for regeneration following nerve injury [114]. It has been suggested that the disruption of the BNB following nerve injury in rats is mediated by Hedgehog pathway inhibition, activation of the innate immunity [113,115] and a crosstalk between Wnt/β-catenin- and Sonic Hedgehog signaling pathways within endoneurial endothelial cells [116]. Therefore, nerve injury-induced neurovascular interactions may contribute to nerve sensitization, allodynia and neuropathic pain development.

### 4.8. The Possible Role of Microorganisms in Peripheral Sensitization

Bacterial infections may induce pain through direct or indirect effects on sensory neurons [117,118,119]. Microorganisms may trigger inflammatory reactions and expose primary afferents to algogenic substances that may drive phenotypic and functional changes in neurons and glia, immune and vascular cells in the peripheral and/or central nervous system [120]. For example, Lipopolysaccharide (LPS), a pro-inflammatory saccharide derived from Gram-negative bacteria, is responsible for the development of critical immune responses via LPS/TLR4 signaling [121]. This persistent, local inflammation-mediated sensitization can lead to chronic peripheral and central sensitization [122,123], which are well-known mechanisms leading to neuropathic pain development [124], in particular when followed by nerve injury. Additionally, LPS-mediated activation of TLR4 signaling can contribute to the disruption of the BNB, which was implicated in PTNP development [115]. Priming with LPS has been shown to induce an early pain-like behavior in rats. Following ION-CCI, mechanical static allodynia and spontaneous pain developed earlier and were more severe in LPS-pretreated rats compared to the control group [120]. Additionally, a significant increase in interleukin 1 beta (IL-1B), a key inflammatory cytokine, was observed in the TG of LPS-pretreated rats compared to controls, and a higher increase in inducible nitric oxide synthase (iNOS) was observed in ipsilateral subnucleus caudalis of LPS-pretreated rats compared to controls [120]. Therefore, inflammatory priming has been suggested to play a key role in the development and maintenance of PTNP implicating IL-1B/iNOS-dependent central sensitization mechanisms. Thus, a pre-existing inflammatory pain could be a risk factor for neuropathic pain development [125]. It is important to note that several studies have demonstrated unique characteristics of P Gingivalis that may play as immunomodulator and may also have palliative effect by increasing levels of anti-inflammatory cytokine IL-10 [126,127]. Local application of P gingivalis reduced pain-like behavior in rats exposed to the Brennan model of pain, and this anti-nociceptive property was at least partially mediated by an increase in IL-10 levels [127].

In summary, injuries at each anatomical level of the sensory system, from the peripheral ending through the sensory ganglia to the spinal cord and central nervous system structures, have been associated with neuropathic pain. Multiple possible sites for the generation of ectopic spontaneous activity have been reported in animal studies. These include along axonal projections and from the cell bodies within sensory ganglia [68,69,71,72,128]. Secondary to nerve injury, different types of cells, such as immune cells, glia and neurons, are affected at each of these anatomical levels, contributing to neuropathic pain development [129,130,131,132]. These cellular constituents interact with each other via gap junctions, synaptic transmission and cell-to-cell signaling [133,134,135]. Following nerve injury, the interactions between the different cellular components are enhanced, forming an excitatory microenvironment that may facilitate spontaneous activity [82]. Additionally, the phenotypic switching of Aβ fibers and their ectopic discharge contribute to neuropathic pain and the sensory changes associated with it. Additionally, nerve injury induced neurovascular interactions and blood–nerve-barrier disruption may contribute to nerve sensitization, allodynia and neuropathic pain development. Finally, bacteria-mediated or pre-existing inflammation may play a key role in the development and maintenance of PTNP.

### 4.9. The Role of Oxytocin in the Pathophysiology of PTNP

Oxytocin (OXT) is a neuropeptide hormone synthesized and secreted by hypothalamic neurons. Direct administration of OXT into the TG attenuated orofacial mechanical hypersensitivity following trigeminal nerve injury [136]. OXT treatment was shown to significantly increase *Ik* current in the TG neurons under neuropathic conditions, suggesting that OXT may promote the functional or expressional recovery of potassium channels involved in hypersensitivity induced by nerve injury [136]. Additionally, coadministration of vasopressin-1A receptor (V1A-R) antagonist entirely abolished the analgesic effects of OXT on nerve injury-induced hypersensitivity [136]. These findings suggest that OXT could interact with V1A-R, which is upregulated following nerve injury and with certain K^+^ channels, which are also functionally changed after nerve injury to exert its analgesic effects. Results from yet another study suggested that OXT application to the nerve-injured site attenuated ION injury-induced mechanical hypersensitivity by inhibition of the TRPV1 and TRPV4 expression in neurons innervating the whisker pad skin [137]. Finally, it has been shown that administration of oxytocin into rats’ TG-activated oxytocin receptors, attenuated orofacial ectopic pain and inhibited the upregulation of OXTR, calcitonin gene-related peptide (CGRP), IL-1B and TNFa in the TG as well as at the spinal trigeminal nucleus caudalis (SpVc) of rats exposed to inferior alveolar nerve transection [138]. These findings suggest that oxytocin may exert analgesic effect by suppressing the inflammatory response in the TG and SpVc of rats exposed to trigeminal nerve injury.

### 4.10. The Role of MicroRNAs in Pathophysiology of PTNP

MicroRNAs (miRNAs) are a class of small noncoding RNAs (approximately 22 nucleotides in length) that participate in RNA silencing and post-transcriptionally regulate gene expression. They play key roles in physiological and pathological processes [139]. MicroRNAs (miRs) are increasingly recognized as potential regulators of neuropathic pain including trigeminal neuropathic pain as they significantly regulate both immune and neuronal processes [140]. Expression levels of miR-146b-5p, −384, 155-5p, and −132-3p are markedly higher in the serum of TN patients than in healthy controls [2,141]. Additionally, MiR-186 suppresses neuroinflammation and neuropathic pain in prosopalgia mice by negatively controlling the NLRP3 inflammasome signaling [142]. Furthermore, expression of miRNA-32-5p was shown to be downregulated in the TG in response to trigeminal nerve injury in rats and promoted the development of neuropathic pain through regulation of Cav3.2 channels [143]. On the other hand, the expression of miR-195 was increased in the caudal brain stems of rats exposed to ION-CCI and its silencing alleviated trigeminal neuropathic pain by inhibiting Sonic Hedgehog (Ssh) signaling activation [55,144]. The observed upregulation of miR-195 was linked to a decreased expression of Patched1, which is the major receptor of the Sonic Hedgehog (Shh) signaling pathway [144]. These findings suggest that miR-195 is involved in the development of nerve injury induced trigeminal neuropathic pain by targeting Patched1 in the Shh signaling pathway and therefore regulating extracellular glutamate [144]. Dysregulation of microRNAs in traumatic neuropathies has also been suggested to contribute to impairment of the blood–nerve barrier (BNB) in nerve injury. For example, expression of microRNA-21-5p (miR-21) was elevated in plasma of patients with complex regional pain syndrome and in nerves of mice exposed to nerve injury. Mice presented with BNB leakage and loss of claudin-1 in injured and spared nerves [145]. Additionally, *RECK* gene, a putative target of miR-21, was decreased, as was downstream *Mmp9* and *Tgfb* was upregulated in the nerves. These findings suggest that the effect of miR-21 on pain-like behavior are exerted via the inhibition of REC, the rise in MMP9 and, ultimately, claudin-1 downregulation [145]. Therefore, miR-21 was proposed to play a major role in neuropathic pain and affects pain-like behavior through different pathways, including inflammation-independent barrier impairment. Another microRNA, mir-223-3p, was shown to alleviate trigeminal neuropathic pain in male mice exposed to ION-CCI by targeting MKNK2 to suppress MAPK/ERK signaling [146].

In summary, an emerging body of evidence supports the role of micro RNAs in the pathophysiology of nerve injury-induced trigeminal neuropathic pain such as PTNP, either through direct regulation of target genes or via inflammation-independent blood–nerve barrier impairment. Taken together, the available evidence suggests that complex reciprocal neuro-immune interactions occur at both the systemic and local levels, in which miRNAs may play an active modulating role and contribute to the pathophysiology of PTNP.

### 4.11. Ganglionic Sensitization and Phenotypic Changes

**Figure 2:**
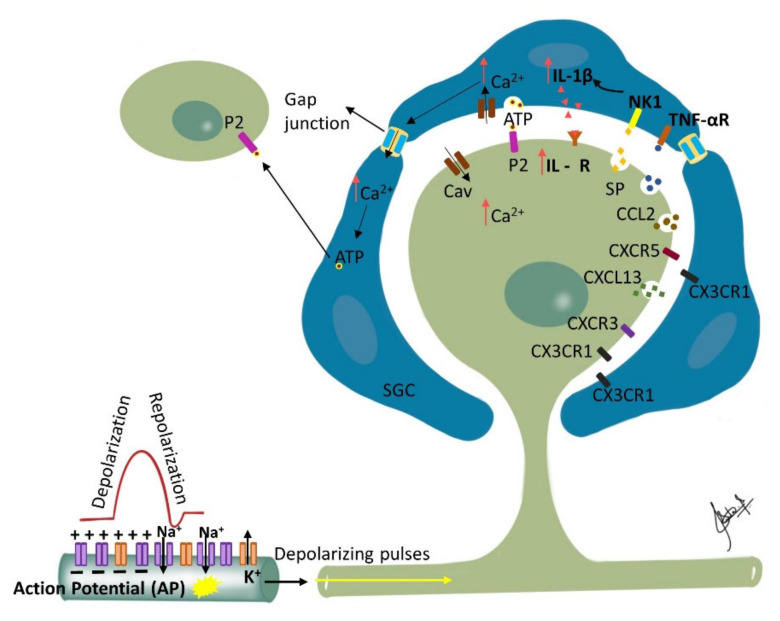
Ganglionic Sensitization: Changes associated with trigeminal nerve injury or inflammation lead to alterations in the cell bodies in the trigeminal ganglion (TG) that start to release pronociceptive substances. Satellite glial cells (SGC) surrounding the TG become activated in the presence of peripheral inflammation that leads to enhanced production and subsequent activation of neurokinin 1 (NK1) receptors with the SP released from the TG nociceptor. Activated SGC produce proinflammatory cytokines (IL-1β, TNF-α) that bind to their receptors on the nociceptor contributing to inflammatory hyperalgesia. Additionally, binding of the somatically released ATP to their corresponding purinergic receptors on SGC leads to increase in intracellular calcium ion concentration which propagates through gap junctions inducing release of ATP by neighboring SGC. The released ATP then binds to purinergic receptors (P2) on neighboring neurons not directly affected by the injury, and affects their excitability. Additionally, released chemokines such as CXCL13 can contribute to neuropathic pain via CXCR5/ERK mediated proinflammatory cytokine production. Furthermore, CXCL10 acts on CXCR3 to induce ERK and AKT activation in TG neurons and contributes to the maintenance of trigeminal neuropathic pain.

Nerve injury induced phenotypic changes in satellite glial cells (SGCs) surrounding the TG [147]. Trigeminal nerve injury or inflammation induces alterations in excitability and molecular expression of voltage-gated ion channels in the TG, which causes hyperexcitability. An influx of inflammatory mediators including pain-associated molecules in the TG neuronal soma occurs by primary afferents, causing changes in intra-ganglionic communication contributing to development and maintenance of neuropathic pain [148]. SGC surrounding the TG become activated in the presence of peripheral inflammation that leads to the enhanced production and subsequent activation of neurokinin 1 (NK1) receptors with the SP released from the TG nociceptor. Membrane depolarization of the TG nociceptors is caused by the activated SGC through the enhanced production of proinflammatory cytokines (IL-1β, TNF-α) and the activation of their receptors on the nociceptor membrane. This process is reported to contribute to central sensitization and resulting allodynia and hyperalgesia [117,149,150,151,152]. Furthermore, neighboring Aβ-TG neurons are activated by the released SP, resulting in the development of mechanical allodynia by modulating their excitability [153] (Figure 2).

The change in the ganglion includes upregulation of ganglion SGCs, and up or down regulation of signaling processes/inflammatory agents/chemical mediators released in ganglion from SGCs, neuronal cells, immune cells and macrophages [67]. Proliferation of SGCs has been shown to peak at approximately 4 days post-injury and dividing SGCs were shown to preferentially surround neurons that were immunopositive for ATF-3 (a marker of nerve injury). Furthermore, nerve injury has been associated with an increase in the expression of glial fibrillary acidic protein (GFAP) in SGSs surrounding ATF-3 immunopositive and immunonegative neurons throughout the ganglia. Additionally, following nerve injury, SGCs express non-glial proteins such as CD45 and CD163, which label resident macrophages and circulating leukocytes, respectively [154]. The silencing of SGC-specific molecules such as Connexin 43, which is involved in intercellular transport or glutamate synthase (involved in glutamate recycling), can dramatically alter nociceptive responses of normal and nerve-injured rats [147]. Finally, studies investigating glutamatergic transmission within the sensory ganglia of rats showed that the soma of primary sensory neurons release glutamate when depolarized and that the somata of primary sensory neurons as well as SGCs express functional glutamate receptors at their surface [155]. Therefore, released glutamate may function as a neuroglial transmitter within the sensory ganglia. These findings suggest that glutamatergic transmission within the ganglion could impact nociceptive thresholds and that SGCs may play an important role in the genesis or maintenance of neuropathic pain. The communication between different cells in the trigeminal ganglion, including between neurons, neurons to SGCs and between the SGCs, is responsible for modulation of neuronal excitability and may play a crucial role in the clinical manifestation of sensory dysfunctions such as ectopic pain in orofacial neuropathic pain [135,153,156,157,158]. This makes the sensory ganglion function as the first relay point of pathophysiological alterations modulating afferent signals and triggering sensitization in the second order neurons and central nervous system [151,159].

### 4.12. Phenotypic Changes and Allodynia

Alterations in the expression of neuropeptides in the TG indicate that functional modifications occur secondary to nerve injury. Due to inflammation, a phenotypic shift is seen in the TG Aβ fibers, which innervates the affected area, as they start to express SP (like C fibers) [39,42,160]. Aβ fibers that transmit innocuous stimuli in healthy tissue acquire the ability to induce nociception due to peripheral stimulation, explaining allodynia. As mentioned previously, SP released by activated TG nociceptors upregulates the expression of NK1 receptors in the medium- and large-diameter neurons in the TG, altering their excitability [161,162]. This depolarization of the resting membrane potential results in an increase in SA [163]. The released SP acts as a diffusible chemical messenger, since it is released non-synaptically, mediating excitability of non-nociceptors in TG neuron, causing mechanical allodynia [161,164].

### 4.13. Response of the Injured Trigeminal System to Stress

Neuropathic pain can be exacerbated in stress and anxiety by the hyperexcitability of sympathetic nervous system activity. Upregulation of α adrenoreceptors at the site of injury and the dorsal root ganglion (DRG) that induces sensitivity to circulating catecholamines is a potential mechanism of this phenomenon. Sympathetic fiber sprouting around the neuronal cell bodies within DRG, which augments sensory–sympathetic interactions, occurs. This phenomenon is exclusive to the spinal system and has not been reported in trigeminal nerve injury, possibly explaining relatively rare sympathetically maintained orofacial pain [21,165,166].

## 5. Conclusions

This review has outlined the current literature in the peripheral cascade of events involved in the pathophysiology of pain due to trigeminal nerve injury, including its clinical presentations. Branches of the trigeminal nerve can be injured as a result of trauma or iatrogenic damage during dental treatments. The current understanding of pathophysiology in PTNP is derived mainly from animal research. Unfortunately, most of the research is limited to spinal nervous system injury models. With the known differences between spinal and trigeminal systems in nerve injury models, it is important to encourage more research in the field of neuropathic orofacial pain focusing on trigeminal nerve injury. Amongst neuropathic trigeminal pain, most studies focus on trigeminal neuralgia models, which differ in pathophysiology, and clinical features from PTNP. It is essential that future therapeutic approaches for patients with PTNP are based on studies that are based on nerve injury to the trigeminal system.
